# Mitochondrial Regulation of the Hypoxia-Inducible Factor in the Development of Pulmonary Hypertension

**DOI:** 10.3390/jcm11175219

**Published:** 2022-09-03

**Authors:** Esraa M. Zeidan, Mohammad Akbar Hossain, Mahmoud El-Daly, Mohammed A. S. Abourehab, Mohamed M. A. Khalifa, Ashraf Taye

**Affiliations:** 1 Department of Pharmacology and Toxicology, Faculty of Pharmacy, Minia University, Minia 61519, Egypt; 2 Department of Pharmacology and Toxicology, Faculty of Medicine at Al Qunfudah, Umm Al Qura University, Al Qunfudah 77207, Saudi Arabia; 3 Department of Pharmaceutics, Faculty of Pharmacy, Umm Al-Qura University, Makkah 21955, Saudi Arabia; 4 Department of Pharmaceutics, Faculty of Pharmacy, Minia University, Minia 61519, Egypt; 5 Department of Pharmacology and Toxicology, Faculty of Pharmacy, South Valley University, Qena 83523, Egypt

**Keywords:** HIF, hypoxia-inducible factor, mitochondria, pulmonary hypertension pathogenesis

## Abstract

Pulmonary hypertension (PH) is a severe progressive lung disorder characterized by pulmonary vasoconstriction and vascular remodeling, culminating in right-sided heart failure and increased mortality. Data from animal models and human subjects demonstrated that hypoxia-inducible factor (HIF)-related signaling is essential in the progression of PH. This review summarizes the regulatory pathways and mechanisms of HIF-mediated signaling, emphasizing the role of mitochondria in HIF regulation and PH pathogenesis. We also try to determine the potential to therapeutically target the components of the HIF system for the management of PH.

## 1. Introduction

Pulmonary hypertension (PH) is a chronic multifactorial pulmonary vascular disease (PVD), which presents in five different categories based on its characteristics [1,2]. According to the 6th World Symposium on Pulmonary Hypertension (WSPH) recommendations, two criteria are essential for the diagnosis of PH: mean pulmonary artery pressure (mPAP) higher than 20 mmHg and increased peripheral vascular resistance (PVR) ≥3 Wood Units (WU) [3]. A direct correlation exists between the product of PVR and cardiac output (CO), and the difference between mPAP and the pulmonary arterial wedge pressure (PAWP) so that PVR × CO = (mPAP − PAWP) [3]. Thus, the inclusion of PVR in the definition of pre-capillary PH is a crucial indicator of pulmonary vascular disease (PVD), as it accounts for the interplay between CO, mPAP, and PAWP.

Rare forms of PH include group I PH, pulmonary arterial hypertension (PAH), and group IV PH due to obstruction of the pulmonary artery, primarily as a result of chronic thromboembolic PH (CTEPH), while the more common forms of PH include group II PH due to left-sided heart failure, group III PH caused by chronic lung disease and/or hypoxia, and group V PH precipitated by other miscellaneous factors [3,4]. Regardless of the etiology, the main characteristics of PH are vasoconstriction and severe remodeling of pulmonary vessels. The features of pulmonary vascular remodeling include thickening of the media, hyperproliferation of vascular cells, enhanced muscularity, increased migration, and increased inflammatory cell recruitment [5,6,7].

Exposure of the alveoli to regional hypoxia constricts the pulmonary vasculature to compensate for the diminished tissue perfusion and maintain adequate arterial oxygenation, a phenomenon known as hypoxic pulmonary vasoconstriction (HPV) [4,8,9,10]. In response to hypoxic stimulation, activating signaling mechanisms in pre-capillary pulmonary arterial smooth muscle cells (PASMCs) is essential to HPV. Moreover, chronic exposure to hypoxia (from hours to days), hence prolonged activation of PASMCs, increases pulmonary vascular resistance (PVR) and contributes to pulmonary hypertension [4,11,12].

Although evidence implicates mitochondrial reactive oxygen species (ROS) in the development of hypoxia-induced PH, possibly via stabilization of the hypoxia-inducible factor-1 (HIF1), the direct role of ROS in the development of PH and thus the therapeutic potential of antioxidant treatment are controversial. Thus, this review focuses on mitochondrial regulation of HIF1α signaling in chronic hypoxia-induced PH.

### Regulation of Hypoxia Inducible Factor (HIF) in Hypoxia

The adaptive mechanisms of vascular cells to chronic hypoxia are orchestrated mainly via the activity of the oxygen-dependent transcription factors known as hypoxia-inducible factors (HIFs) [13]. Since the work published by Wang and colleagues [14], the regulatory role of HIFs on cellular homeostasis in response to hypoxia has been extensively studied [5,15,16,17]. HIF-mediated signaling is now regarded as the cornerstone of oxygen homeostasis that controls multiple hypoxia-responsive genes on the transcriptional level. HIF heterodimers comprise α and β subunits; both are transcription factors of the basic helix–loop–helix PAS family [14]. We now understand that the β subunit (HIFβ) is constitutively expressed, independent of cellular oxygen availability, while the α subunits, e.g., HIF1α, HIF2α, and HIF3α, constitute the oxygen-sensitive element of HIF [14,18,19]. Moreover, under normal tissue oxygenation, the α subunit is extensively hydroxylated and mostly unstable in cells, which is wholly reversed under hypoxia [14,20].

**Figure 1:**
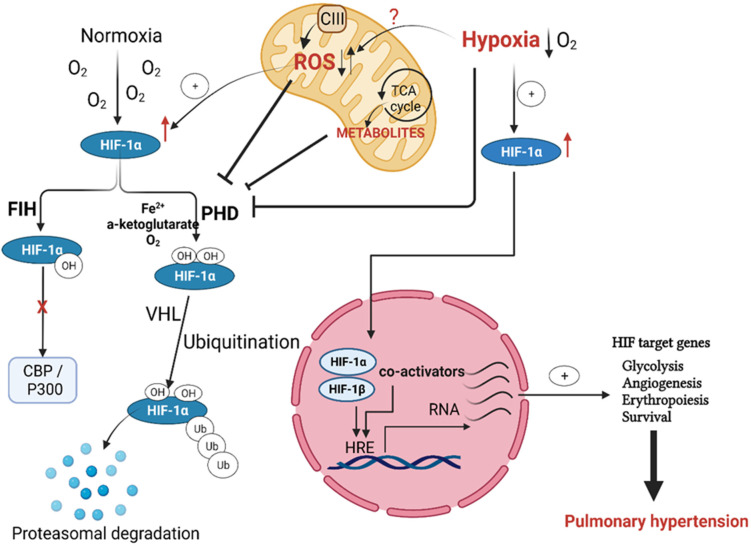
Regulation of hypoxia-inducible factor (HIF) activation in pulmonary hypertension. Both prolyl hydroxylases (PHD) and von Hipple–Lindau (VHL) hydroxylate HIF1α under normoxia, with subsequent targeting for proteasomal degradation. Factor-inhibiting HIF (FIH) controls HIF transactivation by impeding cofactor binding. Hypoxia, as well as mitochondria-produced ROS and metabolites, prevents HIF1 hydroxylation by PHD and subsequently allows its stabilization and nuclear translocation to form the active HIF transcription complex, which binds to its specific hypoxia response elements (HREs). The binding of this transcriptional complex to HREs activates HIF target genes essential to pulmonary hypertension initiation and progression.

The prolyl hydroxylase domain proteins (PHDs), active under a normoxic setting, label the HIFα subunit for degradation by the proteasome after hydroxylation of the proline amino acid residues present in the oxygen-dependent degradation (ODD) domain. PHDs use molecular oxygen (O_2_) as a co-substrate with Fe^2+^ cation and 2-oxoglutarate to hydroxylate HIFα, which is then ubiquitinated via the ligase complex enzyme von Hippel–Lindau (VHL), a well-known tumor suppressor protein. Thus, the PHDs, along with HIF, work as an efficient cellular oxygen sensor that is switched off in hypoxic states and dictates HIFα stabilization and its dimerization with HIFβ, or its proteasomal degradation under normoxia [21,22] (Figure 1). Previous studies showed that HIF1α remains stable upon deletion of the ODD domain even in normoxic cells, which favors its dimerization with the β subunit and drives HIF-dependent signaling, independent of the O_2_ level [23,24].

Although PHDs-mediated hydroxylation of the ODD domain is the primary determinant of HIF fate, deactivation of HIF-mediated gene transcription is also achieved when the asparagine residue (Asp803) of the HIFα subunit C-terminal transactivation domain is hydroxylated. The enzymatic activity of the factor inhibiting HIF (FIH) is responsible for this asparagine hydroxylation. Rather than the prolyl hydroxylation-facilitated proteasomal degradation, hydroxylation of Asp803 by FIH alters the conformation of HIF and prevents its binding with the transcriptional co-activators CBP and p300 [25,26] (Figure 1).

As in hypoxia, reduced oxygen availability inhibits the HIFα subunit hydroxylation, leading to its accumulation, further translocation into the nucleus, and dimerization with the nuclear β subunit forming a functional transcription unit with the transcriptional co-activators CBP and p300. This functional transcription complex binds to the hypoxia response elements (HREs) and allows the expression of hypoxia-specific genes [27,28].

The activated HIF protein starts the adaptive response to hypoxia by activating multiple HIF target genes. This cellular response to hypoxia activates signaling pathways leading to cell survival, proliferation, and metabolic modulations such as repression of mitochondria respiration [29,30]. These HIF-mediated signals augment angiogenesis and erythropoiesis on the tissue or organism level [6,25,31] (Figure 1). Of the HIF target genes, erythropoietin (EPO) and the vascular endothelial growth factor (VEGF) are the primary mediators of the later effects. EPO is a glycoprotein hormone responsible for forming erythrocytes from their precursors via activating its EPO receptors, while VEGF is a primary stimulator of angiogenesis. These effects of EPO and VEGF improve oxygen supply to hypoxic tissue areas in the long term [30].

Besides hypoxia, previous studies showed the activation of HIF1 by non-hypoxic conditions, such as the mechanical effects of vasoconstrictors and signaling events activated by growth factors, cytokines, or hormones, which are upregulated in PH and induce transcription of HIF1α protein [32,33]. Moreover, the dysfunction of succinate dehydrogenase and α-ketoglutarate dehydrogenase, two key enzymes of the Krebs cycle, stabilized HIF1α by accumulating fumarate and succinate that inhibit PHDs [34,35].

Interestingly, HIF1α and HIF2α share a high level of structural similarity, whereas the HIF3α amino acid sequence is quite distinct. However, the three HIFα isoforms have similar O_2_-dependent regulation but differ in tissue expression and abundance [28]. HIF1α is expressed in most cell types, HIF2α can be found chiefly in the vascular endothelium and type II pneumocyte, and HIF3α is present in the cortex, heart, hippocampus, liver, lung, and kidney. Nevertheless, all HIFα subunits are subject to the exact degradation mechanisms [6,28].

## 2. HIF Signaling in Hypoxia-Induced PH

**Figure 2:**
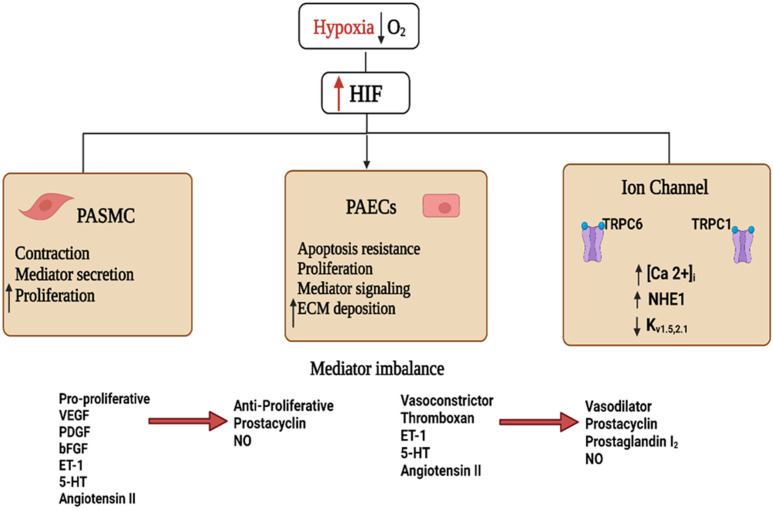
Schematic diagram showing the hypoxia-inducible factor 1 (HIF1)-activated mechanisms involved in pulmonary hypertension. Decreased blood O_2_ partial pressure (↓pO_2_ = hypoxia) acts as a signal in the pulmonary vasculature. Increased HIF stability and signaling during hypoxia activate cellular pathways culminating in vascular endothelial and smooth muscle dysfunction. These effects include increased secretion of vaso-modulators and extracellular matrix (ECM), depolarization of pulmonary artery vascular smooth muscle cells (PAVSMCs) via reduction of outward K^+^ and enhanced inward Ca^2+^ currents, and upregulated Na^+^/H^+^ exchanger isoform 1 (NHE1) function, increasing [Ca^2+^]i, contractility, and proliferation of PAVSMCs. The imbalance between vasodilator and vasoconstrictor signals and the altered cell proliferation orchestrates the pathological signaling events leading to vascular remodeling and the development of pulmonary hypertension. Abbreviations: bFGF, basic fibroblast growth factor; ET-1, endothelin 1; 5-HT, serotonin; NO, nitric oxide; PDGF, platelet-derived growth factor; PASMCS, pulmonary artery smooth muscle cells; PAECs, pulmonary artery endothelial cells; VEGF, vascular endothelial growth factor.

Upon extended exposure to low oxygen levels (chronic hypoxia), HIF isoforms activate the transcription of a variety of genes (Figure 2) that regulate the metabolism and proliferation of pulmonary vascular cells, blood vessel tone, and angiogenesis [18]. Previous studies showed that changes in the PASMCs are the critical processes of PH development after hypoxia exposure [36,37].

The role of HIF1α in altering pulmonary vascular contractility and remodeling has been studied extensively in mice with smooth muscle cells (SMCs)-specific HIF1α deletion. Specific deletion of HIF1α in SMCs in chronic hypoxic mice reduced pulmonary vascular remodeling and PH. However, hypertrophy of the right ventricle was not altered despite decreased pulmonary arterial pressures [38,39]. In contrast, others demonstrated that in mice exposed to normoxia and hypoxia, complete deletion of HIF1α in PASMCs increased the systolic pressure of the right ventricle and myosin light chain (MLC) phosphorylation. However, these mice showed a pulmonary artery muscularization phenotype comparable to controls. Thus, these results demonstrated the role of HIF1α in regulating vascular tone [40].

When under hypoxic conditions, PASMCs showed increased HIF1α-dependent expression of the micro-RNAs miR-9-1 and miR-9-3 [41] and inhibition of BMP signaling [42], which contributed to cell proliferation. Other HIF-regulated targets included genes that enhance oxidative stress, vascular tone, and mitochondrial fragmentation, as well as the activation of the renin-angiotensin-aldosterone system (RAAS) (Figure 2) [6,43].

Exposure of pulmonary artery endothelial cells (PAECs) to chronic hypoxia resulted in various phenotypes, i.e., angiogenesis, migration, and proliferation; HIF isoforms are critical in their identification [44,45,46]. For example, both HIF1/2-mediated signaling events in PAECs alter the expression of several proteins such as glucose transporter 1/3 (GLUT1/3), hexokinase 1/2 (HK1/2), lactate dehydrogenase A (LDHA), and pyruvate dehydrogenase kinase 1 (PDK1) to regulate anaerobic glycolysis [47,48]. Furthermore, HIF2α in isolated PAECs influences hypoxia-induced PH through activating the arginase-1- and arginase-2-dependent pathways, downstream targets for HIF2α, which reduce vascular NO homeostasis, leading to the development of PH in chronic hypoxia-exposed mice. Moreover, loss of arginase-1 in PAECs inhibited the development of PH upon exposing the mice to chronic hypoxia [49].

Moreover, HIF1α is also involved in the contraction of the pulmonary vasculature via its regulation of ion channel expression. In this regard, during hypoxic exposure, HIF1α promoted the expression of the transient receptor potential channel members TRPC1 and TRPC6, leading to elevated PASMCs cytoplasmic Ca^2+^ concentrations, an effect that increases contractility and proliferation of isolated PASMCs (Figure 2) [50,51]. Additionally, hypoxia exposure reduced the expression of the voltage-gated potassium channels Kv1.5 and Kv2.1, causing a reduction in K^+^ current, subsequent membrane depolarization, voltage-gated calcium channel activation, and increased intracellular Ca^2+^. Further evidence on the impact of HIF1α activation in controlling both vascular tone and pulmonary vascular remodeling comes from studies showing that in isolated PASMCs with HIF1α deficiency, there was no decrease in the K^+^ current following exposure to chronic hypoxia [52,53].

Akin to its effect on calcium homeostasis, the role of HIF1α in PH pathogenesis may also involve the dysregulation of pH homeostasis. Consistent with this hypothesis, both hypoxia and hypoxia-induced HIF1α upregulation activate the Na^+^/H^+^ exchanger isoform 1 (NHE1) (Figure 2) [53], leading to alkalinization of the intracellular pH in favor of PASMCs proliferation [28,53]. On the other hand, inhibition of NHE attenuated the development of chronic hypoxia-induced PH and vascular remodeling [54].

Finally, chronic hypoxia exposure causes inflammation as an early consequence and an essential component in developing PH. The targets of HIF activation include pro-inflammatory mediators (e.g., IL-6), nuclear factor kappaB (NF-κB), stromal cell-derived factor-1, and VEGF in PH patients and animal models [43,53,55,56]. Moreover, signaling pathways activated by inflammatory cytokines, such as NF-kB, can further trigger HIF1α activity and potentiate the signaling pathway mTOR/PI3K/Akt, a common downstream inflammation pathway, resulting in HIF stabilization and NF-κB activation [28,31,57]. In addition, these signaling mediators with HIF1α comprise central modulators of the energy metabolism towards aerobic glycolysis, a phenomenon which takes place in both cancer and PAH [58,59,60]. Thus, the ability of inflammatory signals to alter the signaling pathways in the pulmonary vasculature, possibly via initiation of mTOR, NF-κB, HIF signaling, and hypoxia-induced metabolic switch, merits further research.

## 3. Mitochondrial Regulation of HIF in Hypoxia-Induced PH

Mitochondria are essential as signaling organelles for initiating and spreading several homeostatic mechanisms. A critical aspect of mitochondria regulation comprises a dynamic network that undergoes constant merging (fusion) and fragmenting (fission). The GTPases mitofusin-1 and mitofusin-2 control the merging process. In contrast, dynamin-related protein-1 (DRP1) and fission-1 regulate mitochondrial fragmentation [61]. Interestingly, PASMCs isolated from PAH patients showed excessive mitochondrial fragmentation, primarily due to activated DRP1, which translocates to the mitochondria, multimerizes, and induces fission [62]. The activity of DRP1 is controlled by two mitochondrial dynamic proteins: MiD49 and MiD51. Epigenetic activation of MiDs enhances mitotic fission, promoting pathologic cellular proliferation and resistance to apoptosis. Other studies reported elevated expression of MiD49 and MiD51 in PASMCs from human PAH patients or rodent models of PAH induced by monocrotaline (MCT) or Sugen/hypoxia [63]. Moreover, stabilization of HIF1α by CoCl_2_ in lung sections or normal PASMC resulted in DRP1-mediated mitochondrial fission. On the other hand, inhibiting HIF1α decreased DRP1 activation, mitochondrial fragmentation, and PASMC proliferation [62]. These findings proved that HIF1α activation mediates mitochondrial fragmentation resulting in enhanced cell proliferation.

The development of PH involves increased reactive oxygen species (ROS) generated from the mitochondria, and other enzymatic sources, of which the NADPH oxidases (NOXs) are significant players [58]. In mitochondria, the electron transport chain (ETC) complexes are the primary consumers of cellular oxygen, which is critical for ATP production [29,64]. Functioning mitochondria are mandatory for biosynthetic and bioenergetic pathways controlled by the Krebs cycle and mitochondrial membrane potential, respectively [65]. Thus, mitochondria are vital to cellular oxygen-sensing and adaptation to hypoxia of the cell and organism.

Mitochondrial ROS (mtROS) controls the vascular constriction and pulmonary remodeling, accelerating the pathogenesis of PH [30,66]. Accumulating evidence shows that ROS, whether mitochondrial, NOXs-generated, or from other enzymatic sources, are involved in the development of PH and that mtROS may be the upstream ROS source, which is followed by cytosolic NOXs that magnify this signaling paradigm [8,28,66]. Therefore, it was established that there is an intersection between mitochondria-ROS-HIF-Kv channels in the pathogenesis of PAH [58]. Besides, a previous study showed that a genetic abnormality on chromosome-1 resulted in decreased mitochondrial ROS production and consequent activation of HIF1α under normoxic conditions in a rat model of PH [67]. This abnormality inhibited the expression of the oxygen-sensitive, voltage-gated Kv channels (e.g., Kv1.5). Such a change increases membrane depolarization and elevates cytosolic K^+^ and Ca^2+^, favoring a proliferative, apoptosis-resistant PASMC phenotype in PAH [67]. These findings showed that mitochondrial abnormalities and reduced ROS production are upstream of normoxic HIF1α activation, irrespective of PO_2_, creating a pseudo-hypoxic environment analogous to that observed in idiopathic human PAH and the pathophysiological changes in chronically hypoxic rats [67].

In addition, the impairment of the mitochondria ROS–HIF pathway leading to glycolysis and impaired energy metabolism was implicated in PAH and cancer pathogenesis, [58]. Although hypoxia may contribute to mitochondrial dysfunction, the impact of different ROS and mechanisms by which mitochondria-derived ROS (mtROS) promote HIF1α regulation in PH pathogenesis is not clearly defined. There has been significant debate in the literature about whether mitochondrial ROS increases or decreases during acute and chronic hypoxia-induced PH [4,30,37,68,69,70,71,72].

Early reports in the literature showed that mtROS levels decreased due to an acute shortage of oxygen, since molecular O_2_ is an essential substrate for the production of mtROS (Figure 2) [73]. This decline in cellular energy or redox state reduces the production of ROS, which may enhance the ability of cells to sense this drop in the oxygen level [64]. However, others demonstrated the role of mitochondria in sensing the small changes in the oxygen level within the acute oxygen sensing cells [74]. The current understanding is that increased ROS generation in response to hypoxia provokes pulmonary arterial vasoconstriction, hence its importance in the early response to hypoxic changes [68,69,75,76].

Superoxide generation involves multiple sources; however, there is no current consensus on the main pathway(s) involved in its generation during hypoxia. Superoxide radicals, being highly unstable, are converted via the superoxide dismutase SOD enzyme into hydrogen peroxide, which primarily acts as an efficient signaling molecule because it crosses the cell membranes [77,78,79]. Nonetheless, acute hypoxia-induced superoxide generation requires the specific activity of cytochrome c oxidase subunit 4 (Cox4i2); its production hyperpolarizes the mitochondrial membrane. The latter effect enhances the ETC’s superoxide output of complex I (or III) [11].

The role of mitochondrial superoxide as a source of H_2_O_2_ that modulates HPV mediated by the activity of PASMCs in response to hypoxia has been extensively studied [11,26,68]. Evidence on the importance of ETC proteins in ROS generation comes from in vivo studies of mice with PASMCs-specific conditional deletion of the Rieske iron-sulfur protein (RISP), a protein of the mitochondrial ETC Complex III. These mice showed significantly reduced generation of mtROS and inhibition of calcium ion influx in response to hypoxia [80,81]. Nevertheless, in vitro treatment with H_2_O_2_ of pulmonary arteries isolated from these mice resulted in concentration-dependent contractions, confirming that mtROS plays a role in acute hypoxia-induced vasoconstriction [81].

Many researchers studied acute compared to chronic hypoxia effects on vascular function. For example, [71] showed that during acute hypoxia, both cellular and mitochondrial ROS levels were elevated in PASMCs, while, after chronic hypoxia, ROS levels increased in the right ventricle and decreased in PASMCs. Thus, under acute hypoxia exposure, mitochondria contribute to the PASMCs vasoconstriction response through the generation of mtROS, metabolite-induced Ca^2+^ accumulation, and subsequent myosin light chain MLC phosphorylation in specialized oxygen-sensitive cells [71]. In other words, the mitochondrial response to acutely reduced O_2_ levels via sending signals to the sarcoplasmic and plasma membrane, via modulation of ion channels, such as TRPCs, voltage-gated K channels, and L-type calcium channels, culminated in elevated [Ca^2+^]i and potentiated vasoconstriction [30].

Although it is clear that chronic hypoxia modulates mtROS level, contradicting reports showed both increased [82] as well as decreased cellular ROS presentation (Figure 2) [58]. Under chronic hypoxia, the elevated mitochondrial H_2_O_2_ may contribute to the initiation and progression of pulmonary hypertension [82]. In contrast, the results of a study that employed advanced analytical fluorescence spectroscopy techniques for determining the levels of mitochondrial ROS, superoxide, and cytosolic H_2_O_2_, has revealed reduced levels of these ROS parameters in the cytosolic and mitochondrial compartments of PASMCs exposed to 1% O_2_ for five days [71]. These results show that total cellular ROS are reduced after chronic hypoxic exposure [71]. Additionally, in PASMCs exposed to chronic hypoxia, there was no change in the level of 8-hydroxyguanosine, an indicator of DNA damage by ROS, thus providing further evidence of the reduced level of cellular ROS during chronic hypoxia [83]. Moreover, oral treatment with the mitochondria-targeted antioxidant MitoQ neither reduced the proliferation of isolated PASMCs nor the progression of chronic hypoxia-induced pulmonary hypertension [71], but this treatment attenuated cardiac remodeling.

Previous research showed the importance of sirtuins, a class of NAD-dependent deacetylases that regulate mitochondrial function, oxidative stress, and inflammation, especially Sirt3, which is essential in the pathogenesis of conditions of oxygen and glucose deprivation through its effect on redox signaling [84]. Interestingly, in a previous study of PH mice model, mice lacking Sirt3 demonstrated amplified left ventricular hypertrophic changes in response to pharmacological signals, an effect attenuated by Sirt3 overexpression [85]. The Sirt3-deficient hearts displayed increased ROS signaling and diminished expression of necessary antioxidant enzymes such as catalase and manganese superoxide dismutase (MnSOD) [85]. The results of other studies, showing increased ROS output and HIF1α stabilization of HIF1 after Sirt3 deletion, support the importance of Sirt3-mediated maintenance of mitochondrial antioxidant levels, particularly MnSOD expression [86,87]. Conversely, in a different study, PASMCs from Sirt3 knockout mice showed unaltered ROS signaling responses in the mitochondria, neither in the matrix nor the other compartments, upon acute hypoxic exposure (1.5% O_2_; 30 min) [88]. However, the sustained hypoxic exposure (1.5% O_2_; 16 h) following Sirt3 deletion resulted in increased ROS signaling only in the mitochondrial matrix, which was not followed by enhancing HIF1α stabilization. In addition, a 30-day in vivo exposure to chronic hypoxia of Sirt3 knockout mice resulted in the development of PH, as in the WT mice [88]. The discrepancy between such results [85,86,87,88] regarding the Sirt3-mediated responses and its regulation of ROS/HIF1α is attributed to the variations in cell type, experimental settings, or the possible genetic modulation, feedback, or compensatory mechanisms in genetically modified settings.

### Downstream Signaling of mtROS in Chronic Hypoxia

Several studies have implicated mtROS in the pathogenesis of chronic hypoxia-induced PH via its stabilization of HIF1α signaling [16,65], while others have demonstrated no such effect [89]. However, previous studies established the effect of mitochondrial function, cellular redox state, and variations in metabolites and ROS on PHD activity [66,90,91,92]. Chronic hypoxia exposure failed to stabilize HIF or activate EPO expression in ETC-depleted Hep3B, further supporting the hypothesis that a functional mitochondrion is essential for HIF stabilization and signaling [69]. Exogenous ROS application was sufficient to activate normoxic HIF stabilization and signaling during hypoxia, which can be inhibited by antioxidant treatment, suggesting that ROS are essential triggers for HIF-mediated events [6,39,66]. Treating ECs from IPAH patients with MnSOD-specific siRNA decreased the expression of MnSOD, paralleled with increased HIF1α and HRE protein expression [93]. Despite a few contradictory reports, these results support the hypothesis that mtROS-induced stabilization of HIF1α is essential to chronic hypoxia-induced PH.

Furthermore, the iron chelator deferoxamine induces HIF signaling due to its inhibition of the PHD2 required for HIF hydroxylation and destabilization. Experiments on RISP-deficient systems with dysfunctional complex III and altered ROS generation at the ETC level have indicated the essential role of complex III in HIF activation via ROS generation [94]. Moreover, targeted mitochondrial DNA deletion demonstrated altered mitochondrial membrane potential, reduced ETC ROS production, and abolished HIF1α stabilization in response to hypoxia [65,90]. Although these cells displayed a dysfunctional TCA cycle, restoring the genes required for mitochondrial membrane potential in such cells restored ROS generation and hypoxic HIF stabilization in response to hypoxia [65].

In addition to mitochondrial ROS, other metabolites have been found to stabilize HIF in different settings. For example, accumulation of succinate and fumarate in cancer cells enhances the stability of HIF and upregulation of HIF-dependent signaling, which was linked to loss-of-function mutations in fumarate hydratase, leading to accumulation of succinate in the cytosol [34,95]. Fumarate and succinate are TCA metabolites that inhibit PHD enzymes leading to enhanced HIF stabilization [34,95]. Further research revealed the physical interactions between succinate or fumarate with PHDs. The results showed the concentration-dependent PHD-inhibitory effects of fumarate and succinate, resulting in enhanced HIF overexpression [96,97]. Succinate activates IL-1b signaling downstream of HIF1 activation, leading to an inflammatory response, while fumarate levels have been associated with NF-κB activation [17]. Such inflammatory responses amplify hypoxia-induced HIF-derived activation of target genes [17].

The activity of PHDs is essential in HIF degradation [23,34]. The current understanding is that multiple overlapping mechanisms can deactivate PHDs during hypoxia. These include shortage of oxygen level, absence of Fe^2+^ cofactors, accumulation of inhibitory metabolites (succinate and fumarate), intracellular cysteine deficiency, and activation of ROS, among other possibilities. Depending on the context and experimental conditions, one of these mechanisms, or maybe more, could be responsible for “oxygen-sensing” in hypoxic settings, leading to mitochondrial modulation of HIF signaling.

Anecdotal evidence showed that mtROS did not significantly trigger HIF1α stabilization [11,98]. The work of Sommer et al. [11] involved exposing WT and Cox4i2-KO mice (lacking the isoform 2 of mitochondrial complex IV subunit 4) to a 4-week-long 10% O_2_ regimen. Their intriguing results showed that knocking-down of Cox4i2, which is responsible for the release of the superoxide mainly at complex III of the ETC, is required for acute hypoxia adaptation but has no effect on HIF1α stabilization or the development of PH following chronic hypoxia [11]. Accordingly, both strains showed equal degrees of the PH characteristic parameters. Besides, upon exposure to a 36 h 1% O_2_ hypoxic challenge, both animal groups showed similar levels of HIF1α stabilization in the isolated PASMCs [11]. Similar findings were obtained recently by studying a model of alternative oxidase (AOX)-overexpressing mouse, in which the mtROS production is attenuated via bypassing mitochondrial complex III [99,100,101]. Surprisingly, chronic hypoxia-induced PH developed equally in both WT and AOX mice, and both genotypes displayed comparable changes in cardiac output, RVSP, pulmonary vascular muscularization, and right ventricular hypertrophy. Moreover, after three days of hypoxia, HIF1α expression was elevated in the PASMCs from both strains [98].

Together, these findings demonstrated that, under certain conditions, HIF1α stabilization was independent of distal mtROS inhibition. This contradiction with other research results could be related to changes in the concentration of oxygen used in different experiments, the duration of hypoxia, or the cell-type-specific processes of HIF1α stabilization. Therefore, the triggering factors and mechanisms of HIF1α signaling pathways still need further investigation to understand the differential modulation of HIF1α stabilization via mtROS during chronic hypoxia-induced PH; such valuable research will allow the discovery of novel therapeutic approaches.

## 4. Targeting HIF as a Potential Therapeutic Strategy in Pulmonary Hypertension

Many investigated compounds have now been utilized in the preclinical stage to assess their ability to target the HIF pathway therapeutically and determine hypoxic responses in the lung [22]. Most of these compounds showed promising results in reversing or inhibiting the incidence of PH in experimental models (Table 1).

Multiple studies have specifically targeted PHD2, HIF1α, or HIF2α as components of the HIF pathway, with pharmacological compounds in models of PH. These inhibitors, given by various routes of administration, were found to render and reverse PH in different rodent models of PH (hypoxia, MCT, and SuHx) [27]. For example, camptothecin and topotecan deactivate HIF1 at the level of mRNA expression, while celastramycin, 2-methoxyestradiol, and digoxin decrease protein synthesis [103].

In contrast, the YC-1 molecule targets protein accumulation and transcriptional regulation of the HIF axis. Moreover, apigenin and mAb AA98 have altered specific signaling molecules in HIF1 pathway regulation. Additionally, the link between HIF1α, NF-κB, and phosphatidylinositol 3-kinase (PI3K) signaling in PASMCs [114] and other vascular networks continues to gain interest [43]. A previous study demonstrated that caffeic acid phenethyl ester (CAPE), known to inhibit NF-κB, significantly suppressed the AKT/ERK/HIF1α signaling axis in MCT- or chronic hypoxia-induced animal models of PH. This study showed that the CAPE inhibited vascular remodeling by inhibiting HIF1α-induced activation of AKT and ERK signaling. Suppression of those signaling pathways by CAPE inhibited vascular cell proliferation and enhanced the apoptosis of PASMCs [113].

Other potential therapeutics include C76, which suppresses HIF2α at the level of mRNA, heterodimerization, and DNA binding [26]. Notably, C76 showed significant anti-remodeling effects in different animal models of PH [27,103], which manifested as attenuation of vascular muscularization, right-sided hypertrophy, and PAPs in hypoxia-exposed animals, suggesting that HIF2α inhibition could provide a promising therapeutic approach to attenuate hypoxia-induced PH [3,15,39]. Thus, therapeutic targeting of this route is receiving increased interest. Iron supplementation is another way to target the HIF system, as PHD activity is iron-dependent. Indeed, iron infusions reduce the rise in pulmonary arterial pressures in response to acute and chronic hypoxia [115,116].

Furthermore, considering the vital role of ROS in the PH pathogenesis, inhibition of mtROS release by the mitochondria-targeted antioxidant MitoQ [71] or via genetic approaches, e.g., AOX overexpression [98] or Cox4i2 disruption [11], showed inhibition of acute hypoxic pulmonary vasoconstriction but not chronic hypoxia-induced PH. Moreover, incubation with an antioxidant inhibited PH development when administered prior to the hypoxic exposure but not simultaneously [117]. Additionally, in previous animal studies, several antioxidant-rich substances have been utilized to slow the course of PH pathogenesis. Examples include allopurinol, SOD, pyrrolidine dithiocarbamate (PDTC), and sulfur dioxide [118,119]. However, using available non-selective antioxidants may impact the tricky balance that promotes homeostatic redox signaling or causes detrimental oxidative stress. As a result, mitochondria-targeted compounds that inhibit specific molecules implicated in ROS generation, such as RISP in complex III, may present successful PH treatments [80].

## 5. Conclusions

Isoforms of HIF play a critical role in the pathogenesis of hypoxia-induced PH through cellular and molecular signaling modulation that alters vasoconstriction, PASMC proliferation, and angiogenesis. HIF1 and HIF2 exert various and complementary effects upon exposure to hypoxia in both pulmonary vascular smooth muscle and endothelial cells. In addition, mitochondria-derived mechanisms promote HIF stabilization with subsequent progression and development of the PH disease. Mitochondria regulate acute and chronic responses to hypoxia exposure. Longer-term adaptation to hypoxia exposure is mediated by HIF activation via ROS and/or metabolite-induced PHD deactivation. However, the detailed upstream and downstream mechanisms of HIF signaling require further investigation. More research is needed to find new, effective, and selective inhibitors that target specific locations in the HIF pathway and subsequent new therapeutic approaches for PH treatment.

## Figures and Tables

**Table 1:** Preclinical studies that utilized various therapeutic strategies targeting HIF signaling in PH development.

Compound	Experimental Setting	Target	Main Findings	Ref.
**Compound 76 (C76)**	In vitro: PASMCs, PAECs, lung samples from iPAH patients In vivo: MCT and SuHx models of PAH	⇑ IRP1 to inhibit HIF2α signaling	⇓ RVSP ⇓ RVH ⇓ RVR	[102,103]
**3-(5′-hydroxymethyl-2′-furyl)-1-benzylindazole (yc-1)**	In vitro: hPASMCs exposed to hypoxia In vivo: chronic hypoxia (28 day) PH mouse model	⇑ sGC signaling to inhibit HIF1α expression	⇓ PVR ⇓ RVH	[104]
**Topotecan (TPT)**	In vitro: hPASMCs exposed to hypoxia In vivo: chronic hypoxia (28 day) PH rat model PH	⇓ HIF1α protein accumulation. ⇓ HIF1α target genes expression	⇓ PASMCs growth. ⇓ PVR	[105]
**Celastramycin**	In vitro: PASMCs from iPAH patients.	⇓ HIF1α	⇓ PASMCs growth. ⇓ oxidative stress and inflammation.	[106]
**2-methoxyestradiol (2-ME_2_)**	In vitro: hPASMCs exposed to hypoxia In vivo: chronic hypoxia (28 day) PH rat model PH	⇑ MnSOD activity ⇓ ROS production ⇓ HIF1α expression	⇓ PASMCs growth ⇓ RVSP ⇓ RVH ⇓ PVR	[107,108]
**Apigenin**	In vivo: chronic hypoxia (28 day) PH rat model PH	⇓ Akt signaling ⇓ HIF1α expression	⇓ RVH ⇓ PVR	[109]
**Digoxin**	In vivo: chronic hypoxia (28 day) PH mouse model PH	⇓ HIF1α transcription and protein synthesis	⇓ RVP ⇓ RVR	[110]
**Anti-CD146 mAb AA98**	In vivo: chronic hypoxia (28 day) PH mouse model PH, and MCT mouse model	⇓ CD146 dimerization and ⇓ HIF1α response	⇑ cardiac function ⇓ PH development	[111]
**PHD2 activator (R59949)**	In vivo: chronic hypoxia (28 day) PH mouse model PH	⇑ PHD2 and ⇓ HIF1α levels	⇓ PVR	[112]
**Caffeic acid phenethyl ester (CAPE)**	In vivo: MCT mouse model	⇓ AKT/ERK activation and ⇓ HIF1α expression	⇓ proliferation & apoptosis resistance. ⇓ RVSP ⇓ PVR	[113]

Abbreviations; AKT, phosphorylated protein kinase B; IRP1, iron-regulatory protein; MCT, monocrotaline; PASMCS, pulmonary artery smooth muscle cells; PAECs, pulmonary artery endothelial cells; PVR, pulmonary vascular remodeling; RVH, right ventricular hypertrophy; RVP, right ventricular pressure; RVR, right-ventricular remodelling; RVSP, right ventricular systolic pressure, SU/HX; sugen/hypoxia.

## Data Availability

Not applicable.
